# C-reactive protein/albumin ratio at ICU discharge as a predictor of post-ICU death: a new useful tool

**DOI:** 10.1186/cc10200

**Published:** 2011-06-22

**Authors:** LCP Azevedo, OT Ranzani, LF Prada, FG Zampieri, JV Pinaffi, LC Battaini, YC Setogute, DN Forte, LC Azevedo, M Park

**Affiliations:** 1 Hospital das Clínicas, Faculdade de Medicina da Universidade de São Paulo, São Paulo - SP, Brazil

## Introduction

There are classical predictors of death after ICU discharge, such as age, severity of disease and level of nursing care. CRP concentrations at discharge have also been reported as a predictor of in-hospital outcome, but with controversial results. Considering that albumin is a negative acute-phase protein and its decrease may be an indicator of disease severity, we hypothesized that the CRP/albumin ratio could be a marker of unfavorable outcomes in the post-ICU period.

## Objective

This study aimed to investigate whether the CRP/albumin ratio at ICU discharge may be a predictor of post-ICU death. We also evaluated which is the best cut-off value of the CRP/albumin ratio to predict mortality.

## Methods

Patients discharged from the ICU after at least 72 hours of stay were retrieved from our prospective collected database. A multivariate analysis was performed using a backward-LR binary logistic model taking in-hospital death as a dependent variable and age, APACHE II at admission, comorbidities, ICU length of stay (LOS), support during ICU, SOFA at ICU discharge, admission characteristics and CRP/albumin ratio as independent variables. ROC curves and the Youden index were used to calculate the best cut-off value of the CRP/albumin ratio.

## Results

We retrieved 548 patients. Mean age was 49 ± 19 years, median APACHE II score at admission was 16 (10 to 21) and median SOFA score at discharge was 2 (1 to 3). The main causes of admission were septic syndromes and respiratory failure. The in-hospital mortality after ICU discharge was 18.6%. The ICU length of stay was 7 (4 to 11) days. At the moment of ICU discharge the median CRP was 47 (22 to 109) mg/l, albumin 27 (23 to 31) g/l and the mean of CRP/albumin ratio was 3. The multivariate analysis resulted in the following independent in-hospital death predictors: age (OR = 1.028, 95% CI = 1.014 to 1.043, *P *< 0.001).

## Conclusion

We demonstrated that the CRP/albumin ratio, a possible marker of residual inflammation, in addition to classical variables, could be a useful and objective tool to support the clinical judgment on the ICU discharge decision process. The best value of the CRP/albumin ratio to predict death after ICU discharge is 2. Further prospective investigations are necessary to confirm these findings.
