# Correction: C-reactive protein/albumin ratio at ICU discharge as a predictor of post-ICU death: a new useful tool

**DOI:** 10.1186/cc10363

**Published:** 2011-09-14

**Authors:** LCP Azevedo, OT Ranzani, LF Prada, FG Zampieri, JV Pinaffi, LC Battaini, YC Setogute, DN Forte, LC Azevedo, M Park

**Affiliations:** 1 Hospital das Clínicas, Faculdade de Medicina da Universidade de São Paulo, São Paulo - SP, Brazil

## Correction

After the publication of this abstract [1], we found that not all data of results section were published. The complete multivariate analysis and the best cut-off value of the CRP/albumin ratio are now published: The multivariate analysis resulted in the follow independent in-hospital death predictors: Age (OR 1.028, 95% CI 1.014-1.043, p = 0< 0.001), comorbitidies (OR 1.247, 95% CI 1.036-1.500, p = 0.020), ICU LOS (OR 1.031, 95% CI 1.003-1.0609, p = 0.029), Septic Syndrome (OR 1.683, 95% CI 1.027-2.758, p = 0.039), SOFA (OR 1.284, 95% CI 1.151-1.432, p < 0.001) and CRP/albumin ratio (OR 1.809, 95% CI 1.099-2.977, p = 0.020). The best value of CRP/albumin ratio to predict death after ICU discharge was 2.
